# Japanese health and safety information for overseas visitors: protocol for a randomized controlled trial

**DOI:** 10.1186/s12889-021-10627-w

**Published:** 2021-03-21

**Authors:** Mariko Nishikawa, Masaaki Yamanaka, Akira Shibanuma, Junko Kiriya, Masamine Jimba

**Affiliations:** 1 Department of Global Health and Nursing, Graduate School of Nursing, The University of Human Environments, Nagoya, 3-220, Ebata cho, Obu City, Aichi 474-0035 Japan; 2 Department of Maritime Science and Technology, Japan Coast Guard Academy, Kure, Japan; 3 Department of Community and Global Health, Graduate School of Medicine, The University of Tokyo, Tokyo, Japan

**Keywords:** CSQ-8, Health education, Overseas visitors, *Sa-Chan* game

## Abstract

**Background:**

Before the COVID-19 pandemic occurred in January 2020, the number of overseas visitors to Japan had increased threefold over the last decade. To minimize the risk of health problems, visitors should be able to access information on the health care systems of the places they visit. Most short-term overseas visitors are young adults. Although they are not very likely to get sick from noncommunicable diseases, they are at high risk for injury and often experience stomach ailments, fever, or nausea when travelling. The objective of this study is to evaluate culturally and linguistically appropriate health information on preventive health behaviours and the health care system in Japan. We will examine the level of satisfaction of overseas visitors to Japan with health care-related educational materials using a five-minute digital game named *Sa-Chan Japan.*

**Methods:**

Our study is a randomized controlled trial (RCT). We will assess both satisfaction and motivation before, during, and after the interventions and examine the changes over time. The intervention group will comprise overseas visitors who will view and answer questions in an animation named *Sa-Chan Japan*. The control group will comprise overseas visitors who will watch an English digital animation named *Mari Info Japan*. We will recruit 1002 participants through the *Macromill* Internet portal. We will contact overseas visitors who have either visited or wish to visit Japan from the United Kingdom, United States, and Australia. The participants will fill out a self-administered questionnaire online in the first quarter of 2021. We will determine the participants’ levels of satisfaction with the CSQ-8 (8-item Client Satisfaction Questionnaire). We will analyse the median score of the overseas visitors with both the Wilcoxon rank-sum and the Wilcoxon signed-rank tests. Our protocol of randomized controlled trials follows the SPIRIT guidelines.

**Discussion:**

Our research will utilize unique digital education strategies in a game that promotes health and safety among overseas visitors to Japan. We believe the results of this study will be useful in overcoming the current challenges regarding pretravel health requirements for overseas visitors worldwide.

**Trial registration:**

Version 1 of this trial was registered in the UMIN-CTR (University Hospital Medical Information Network Center Clinical Trials Registry), and the trial registration data are available on UMIN000042483, November 17, 2020.

## Background

The number of overseas visitors to Japan has steadily increased over the last decade [1] from 8.6 million in 2010 to 31.8 million in 2019 [2]. Notwithstanding the disruption to travel caused by the COVID-19 pandemic, this number will continue to rise due to increasing global tourism, international conferences, and major sporting events [2]. The potential for public health issues among mass gatherings at these large events should be considered [3].

It is imperative that overseas visitors are able to access information about the health care system of the country they are visiting to reduce risks and enjoy a comfortable stay [4]. Wadhwaniya and Hyder [4] examined how overseas visitors obtained information and where they visited. Some of these visitors were immunized at clinics before travelling to developing countries, even though the health risks were not confined to those countries.

There are three main concerns associated with the low level of health information accessed by overseas visitors to Japan. First, overseas visitors tend to be young adults and think they are not very likely to become sick while travelling, but they are at high risk for injury [5]. Only 18% (45 out of 241) of overseas visitors, with a median age of 30–39 years, accessed information about the Japanese health care system in our previous study [6]. As one of the fastest growing host countries, Japan needs to rethink how its health care information will reach overseas visitors, including young adults.

Second, the effectiveness of pretravel health issue prevention is dependent upon the presentation and content of the information [7, 8]. Health information for overseas visitors is usually provided through websites, pamphlets, travel books, or visiting clinics in their home countries [7, 9, 10]. Currently, public health authorities of various countries provide health and safety information. This information is located at disparate places and may be inadequate for certain overseas visitors. Furthermore, much of the information is about infectious diseases and immunizations for developing countries [11–13]. For instance, in our earlier study conducted before COVID-19, we concluded that overseas visitors are most concerned about medical costs, the Japanese language, and informed consent at clinics and hospitals, but there is not enough information to decrease these concerns [6, 14]. Third, although studies have confirmed that educational games are beneficial for sharing health-related information [15, 16], we have not found educational games that provide health-related information for travellers.

Overseas visitors generally consider Japan a developed nation that has a health care system with high standards. However, they do not know how to navigate the Japanese health care system should the need arise. Host nations have an obligation to provide accurate and useful information to overseas visitors about their health care system [17], illness prevention [18, 19] and procedures to access health facilities [14, 17, 20] in an efficient manner so that overseas visitors are not anxious about visiting other nations [4, 21]. Comprehensive and effective health education methods can convey vital information. Advancements in digital technology are driving changes, and information is now provided in several languages and in various formats [22]. These changes benefit most visitors, including young visitors, who are more likely to be at risk of injury when visiting foreign countries.

A digital game is an attractive way to distribute visually and culturally relevant information [23–25]. In a previous study, a digital game on insulin therapy for children with type 1 diabetes was used, and non-supervised usage of the educational game “L’Affaire Birman” was able to improve insulin titration and carbohydrate quantification results [26, 27]. Another game used by general surgery residents in classrooms showed a significant increase in short- and long-term medical knowledge that was retained, with high learner satisfaction [28]. A separate study on lecturing nursing students showed that an educational game was both liked and accepted by the students and considered a satisfying teaching technique [16].

Digital games can also be used to share information on travel health with overseas visitors. In this research, we will evaluate the effect of a five-minute digital game titled *Sa-Chan Japan* (Table 1). We will examine the levels of satisfaction and motivation of overseas visitors to Japan regarding their educational experience.

**Table 1:**
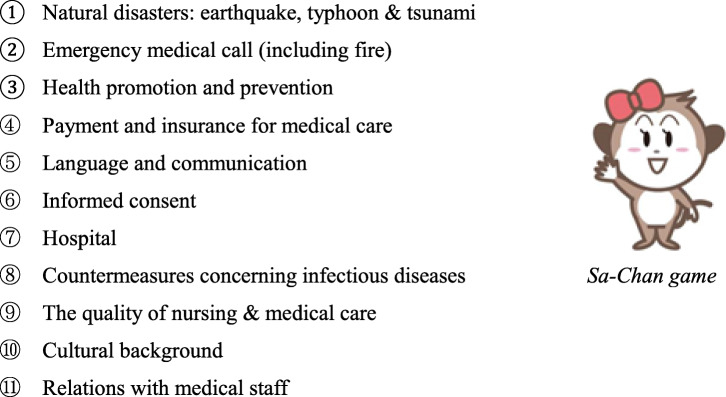
Intervention group

*Sa-chan Game*: Contents of Japanese health & safety game for international visitors

## Methods

### Study design and procedures

**Fig. 1:**
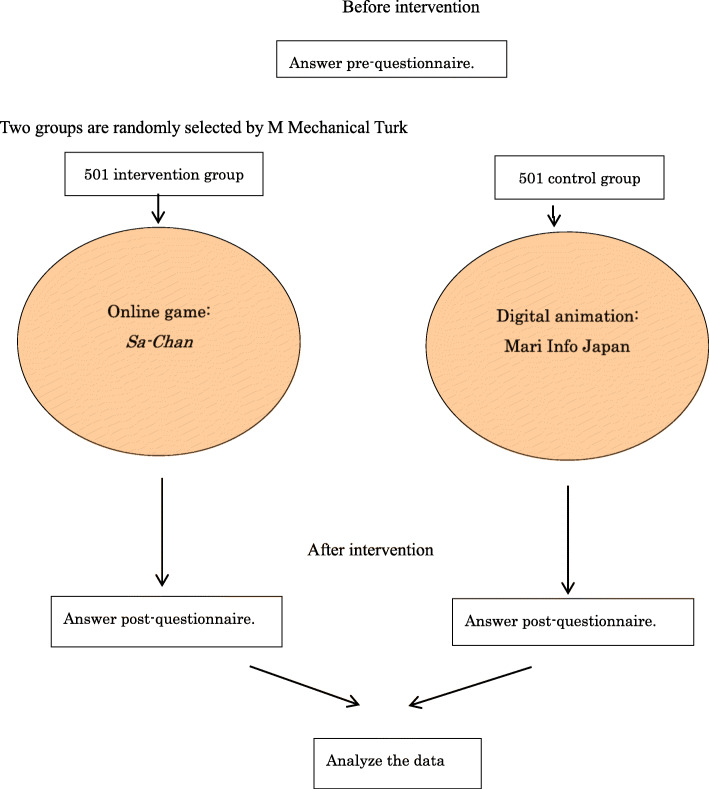
Schematic design of study

We will conduct this randomized controlled trial to examine the efficacy of an animation game in improving both satisfaction and behavioural changes among current and potential visitors to Japan. The participants will complete the survey online. The participants will answer a questionnaire on the satisfaction and behavioural changes prior to and after participating in one of two interventions. We will evaluate the changes in their satisfaction and motivation levels (Fig. 1).

### Participants

#### Sample size

In this study, we expect to recruit 1002 participants via a Macromill internet panel. In the sample size calculation, a 95% confidence level and 80% power were used to detect a difference of 0.178 in the questionnaire score, with a standard deviation of 1.0, extra parameter of 0.0 and alpha of 0.05.

#### Eligibility

Individuals who are planning to visit Japan from the United States, the United Kingdom and Australia will be recruited by a website. They will indicate whether they are willing to participate in research online through a company. The questionnaire will be in English only to prevent biases regarding interpretation. Individuals who are 18 years old or older, understand English, and have previously visited or wish to visit Japan will be considered eligible to ensure the validity of the responses to our questionnaire. We will consider individuals who are interested in the health care services in Japan.

#### Enrolment procedure

We will allocate the participants to either an intervention group or a control group randomly through the Macromill Company services. The participants in this study will be screened by monitors through an online questionnaire. The participants will be able to access the survey site and to receive e-mail notices. The monitors will determine who is enrolled in our study. At the beginning of the questionnaire, the target conditions are explained, and the questionnaire is designed so that only those who meet the conditions stipulated by our research can complete the questionnaire.

The participants will be asked about their satisfaction with the Japanese health care system and health information. We will repeat this procedure until we reach our required sample size. We will also ask them to answer the questions based on their current knowledge and to not search for answers by accessing other websites or references. Each participant will receive a one U.S. dollar, one Australian dollar or one Euro gift certificate from Macromill Company upon completion of the eligibility survey in March 2021.

### Interventions

#### Intervention

The intervention group will watch a five-minute digital game titled *Sa-Chan Game* in English (Table 1). This animation is in the format of a quiz that aims to provide information on the health care system and safety in Japan for overseas visitors. Its content is based on the results of a previous study on overseas visitors’ concerns about visiting Japan [14]. It starts with Asian music and contains 11 items. We will share the animated game through a website.

#### Control intervention

The control group will watch a four-minute digital animation in English named *Mari Info Japan* (Table 2). The aim is to provide information about the health care system in Japan for overseas visitors [14]. It will last for 4 min and contain 11 items in English. We will provide the digital animation in the same manner as for the intervention group though a website.

**Table 2:**
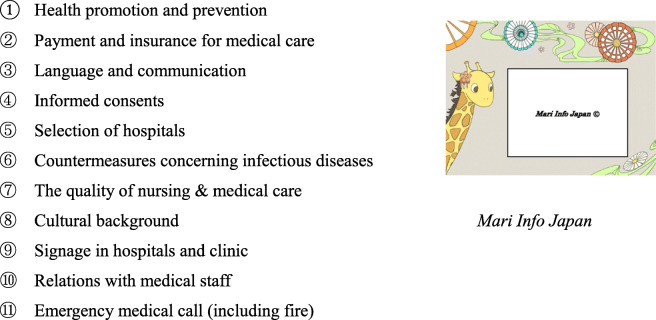
Control group

*Mari Info Japan*: Contents of Japanese health & safety Information for international visitors

### Outcomes

1. The primary outcome of this study is the difference in the average or median CSQ-8 (8-item Client Satisfaction Questionnaire) [29] score between the participants who will have played the *Sa-Chan* game and controls immediately after the interventions. We will assess the outcome using a self-administered questionnaire, the CSQ-8 scale, which has been used widely for health education [29] and has been shown to have reliable and valid in previous research [30]. The CSQ-8 is an eight-item questionnaire that uses a four-point Likert scale and will be used to assess the respondent’s level of satisfaction regarding the health care system and safety in Japan. The total score ranges from 8 to 32. A high score denotes greater satisfaction. The reliability of the questionnaire will be examined by Cronbach’s alpha.
2. The second outcome of the study is the difference in motivation between the participants who will have played the *Sa-Chan* game and the controls immediately after the interventions. We will ask one question, “Are you likely to follow this information yourself”, and the participants will respond using a four-point Likert scale to determine whether they will change their behaviour or assess their level of motivation to follow the Japanese health-related guidelines. We will collect the data before and after the interventions. We will evaluate the data with the Information-Motivation-Behavioural Skills model [31]. This model has been used in a number of risk reduction behaviour studies [32].
3. The third outcome is whether the participants understood the information presented in the *Sa-Chan* game. When a participant understands the information corresponding to each of 15 items, he or she will respond with “yes” to the item. The questions will be related to both interventions. If a participant understands how to deal with the following topics in Japan, he or she might choose correct answers.

In total, the questionnaire that will be used to assess the outcomes in (1), (2), and (3) and performing the intervention will take less than 10 min to complete. The participants will be visitors or individuals who wish to visit Japan.

#### Other information

We will also include in this randomized controlled study basic characteristics of the participants, such as their previous visits to Japan, sex, age, and educational level. We will determine whether the distributions of these characteristics are balance between the two groups and identify factors that might influence the results. We conducted a pilot test with 13 participants at a college in New York on August 12, 2018.

### Bias prevention

The allocation of participants to either the control or the intervention group will be blinded to both the participants and the researchers.

#### Data analysis

1. For the primary outcome of this study, we will analyse the difference in the median scores for the CSQ-8 recorded before and after the intervention between groups with the Wilcoxon signed-rank test. To compare the pre- and post-intervention scores between groups, the Wilcoxon rank-sum test will be used. We will adjust for other potential demographic factors that might affect the results of the multiple regressions.
2. For the secondary outcome, regarding motivation, we will compare the differences in the pre- and postintervention scores for the behavioural change question between groups with the Wilcoxon rank-sum test.
3. The third outcome is related to the participant’s understanding of the *Sa-Chan* game, which includes health knowledge questions. We will determine whether the answers are related to the characteristics of the participants.

All data analyses will be conducted using the JMP statistical package (version 14.0). The answers provided for the open-ended questions about Japanese health information will be examined by word-frequency analysis, with involves a word relationship network and co-occurrence, using the language analysis software Text Mining Studio (version 6.2) [33].

## Discussion

The study will offer a unique digital education strategy in the form of the game *Sa-Chan* to overseas visitors to stay healthy and safe. To welcome visitors from other nations, the host country needs to provide practical and useful information in an attractive and effective manner.

## Data Availability

Not applicable.

## Abbreviations

COVID-19: Coronavirus Disease 2019
CSQ-8: 8-item Client Satisfaction Questionnaire
Trials UMIN-CTR: University Hospital Medical Information Network Center Clinical Trials Registry
RCT: Randomized controlled trial
